# Association and diagnostic value of serum SPINK4 in colorectal cancer

**DOI:** 10.7717/peerj.6679

**Published:** 2019-04-04

**Authors:** Mingzhi Xie, Kezhi Li, Jilin Li, Dongcheng Lu, Bangli Hu

**Affiliations:** 1 Department of Chemotherapy, Affiliated Tumor Hospital of Guangxi Medical University, Nanning, Guangxi, P.R. China; 2 Department of Research, Affiliated Tumor Hospital of Guangxi Medical University, Nanning, Guangxi, P.R. China

**Keywords:** Colorectal cancer, SPINK4, Diagnosis, Prognosis, Serum

## Abstract

The role of serum serine peptidase inhibitor, Kazal type 4 (SPINK4), in colorectal cancer (CRC) is largely unknown. This study aimed to explore the association and diagnostic value of serum SPINK4 in CRC. A total of 70 preoperative CRC patients, 30 postoperative CRC patients, 30 gastric cancer patients, and 30 healthy controls were enrolled. Using enzyme-linked immunosorbent assays, we found that the serum SPINK4 level was significantly increased in preoperative CRC compared with postoperative CRC patients, gastric cancer patients, and healthy controls (*p* < 0.05). The serum SPINK4 level was remarkably elevated in colon cancer compared with rectal cancer and was enhanced in the M1 stage compared with the M0 stage (*p* < 0.05). The area under the receiver operating characteristic curve of serum SPINK4 level in the diagnosis of CRC was 0.9186, with a sensitivity and specificity of 0.886 and 0.900, respectively, and a cut-off value of 2.065. There was no significant difference between high and low expression of serum SPINK4 regarding the overall survival time and disease-free survival (*p* > 0.05). This study demonstrated that the serum SPINK4 level increased in CRC and was associated with the location and distant metastasis of CRC. It had a high diagnostic value in CRC but was not associated with the survival of CRC patients.

## Introduction

Colorectal cancer (CRC) remains a serious public health problem worldwide, being the third most common gastrointestinal cancer (Siegel et al., 2017), and the incidence of CRC is still increasing in China (Chen et al., 2016). China had 245,000 new CRC cases and 139,000 deaths from CRC in 2012, which made it the fifth most common cancer in men and the fourth in women (Gu et al., 2018). Although great advancements have been made in surgical procedures and adjuvant therapies, the overall survival of CRC patients is still poor, especially that of patients at later stages of disease (Fang et al., 2013; Nakajima et al., 2017). However, many CRC patients are initially diagnosed at advanced stages, since early-stage CRC is either asymptomatic or presents with non-specific symptoms (Clarke & Feuerstein, 2018; Hall et al., 2016). Therefore, finding reliable biomarkers associated with the pathogenesis of CRC, and early diagnosis and prognosis of CRC has become imperative for patients.

CRC is a heterogeneous disease of high molecular complexity, and aberrant expression of molecular biomarkers partly contributes to the development and progression of CRC (Bogaert & Prenen, 2014; Dienstmann, Salazar & Tabernero, 2014). Currently, some tumor biomarkers have been applied in the conventional detection of cancer, including carcinoembryonic antigen (CEA), CA125, and CA199. However, none of them had organ specificity because their levels have been shown to increase in many non-neoplastic conditions. In addition, recent studies indicated that these tumor biomarkers were subject to insufficient sensitivity and low prognostic value in CRC because of the highly heterogeneous nature of CRC (Bystrom et al., 2012; Hara et al., 2010; Stojkovic Lalosevic et al., 2017). Moreover, the relationship between these biomarkers and clinical features is also controversial (Dolscheid-Pommerich et al., 2017; Wild et al., 2010), which reduced their diagnostic and prognostic value in CRC. In recent years, a growing number of independent indicators in CRC have been explored, such as serum pentraxin-3 (Liu, Zhao & Guo, 2018), CNPY2 (Peng et al., 2018), and HSP-90*α* (Kasanga et al., 2018), which have been of great help in the diagnosis and prognosis of CRC; however, the reliability of these biomarkers requires further study.

Serum serine peptidase inhibitor, Kazal type 4 (SPINK4), also known as PEC60 or HEL136, was originally isolated from pig intestine and is abundantly expressed in human and porcine goblet cells in the crypts of Lieberkühn; it is also expressed in monocytes and the central nervous system (Wapenaar et al., 2007). *SPINK4* encodes a 86-amino acid long precursor protein containing a 26-amino acid signal sequence. *SPINK4* mRNA expression was elevated in porcine duodenum and showed a strong PEC-60-like immunoreactivity in the cytoplasm of the majority of goblet cells of the epithelium (Metsis et al., 1992). A previous study found that expression of SPINK4 was decreased in colon cancer cells when SPDEF was inhibited and resulted in terminal differentiation and maturation of intestinal goblet cells (Noah et al., 2010), suggesting that SPINK4 was involved in the pathogenesis of colon cancer. To date, there have been no previous reports of the role of SPINK4 in CRC. Thus, the aim of the present study was to measure the serum levels of SPINK4 in CRC, to analyze their correlation with clinical features, and explore the diagnostic and prognostic value in CRC.

## Materials and Methods

### Patients and healthy controls

Serum samples were collected from patients admitted to the Affiliated Tumor Hospital of Guangxi Medical University and Bio-bank of the Tumor Hospital of Guangxi between October 2014 and December 2016. All the cancers were verified by pathological and cytological diagnoses. Healthy controls were selected randomly from the Physical Examination Center of the Affiliated Tumor Hospital of Guangxi Medical University. All patients with autoimmune diseases, cardiovascular diseases, severe liver and kidney diseases, hematologic diseases, and infectious diseases were excluded. This study was conducted in accordance with the ethical guidelines of the 2008 Declaration of Helsinki and approved by the ethics committee of the Affiliated Tumor Hospital of Guangxi Medical University. All participants provided written informed consent.

### Sample collection and follow-up

A 5 mL sample of fasting peripheral blood was collected from each participant. The postoperative sample was collected 1–2 weeks after surgery. Serum samples were obtained by centrifuging at 1,500 g for 10 min at 4 °C and then stored at −80 °C in a 200 µL/tube for further use. The serum samples of healthy controls were collected in the morning. Follow-up was implemented to evaluate the overall survival (OS) and disease-free survival (DFS) time of CRC patients postoperatively.

### Detection of serum tumor biomarkers and SPINK4

Enzyme-linked immunosorbent assay (ELISA) kits (Yuchun, Shanghai, China) were used to measure the serum SPINK4 level. Experiments were performed according to manufacturers’ instructions. Optical density (OD) values were read at a wavelength of 450 nm using a 96-well microplate. The concentration of SPINK4 was calculated based on the standard curve. Serum levels of CEA were tested by chemiluminescence immunoassay, CA19-9 was detected by automatic electrochemiluminescence.

### Statistical analysis

R version 3.4.1 (R Core Team, 2018) was used for all statistical analyses. Data were expressed as the mean ± standard deviation or median and interquartile range as appropriate. The differences among groups were compared via analysis of variance or Student’s *t*-test as appropriate. Nonparametric methods were used to analyze the differences in serum SPINK4 level between CRC patients and controls. Correlation analysis was performed using Spearman’s rank correlation test. Receiver operating characteristic curve analysis was used to evaluate the sensitivity, specificity, and respective areas under the curve (AUCs) with 95% confidence interval (CI) for SPINK4. Survival curves were created using the Kaplan–Meier method, and survival was compared using log-rank tests. A *p*-value <  0.05 was considered statistically significant.

## Results

### Demographics of participants

**Figure 1:**
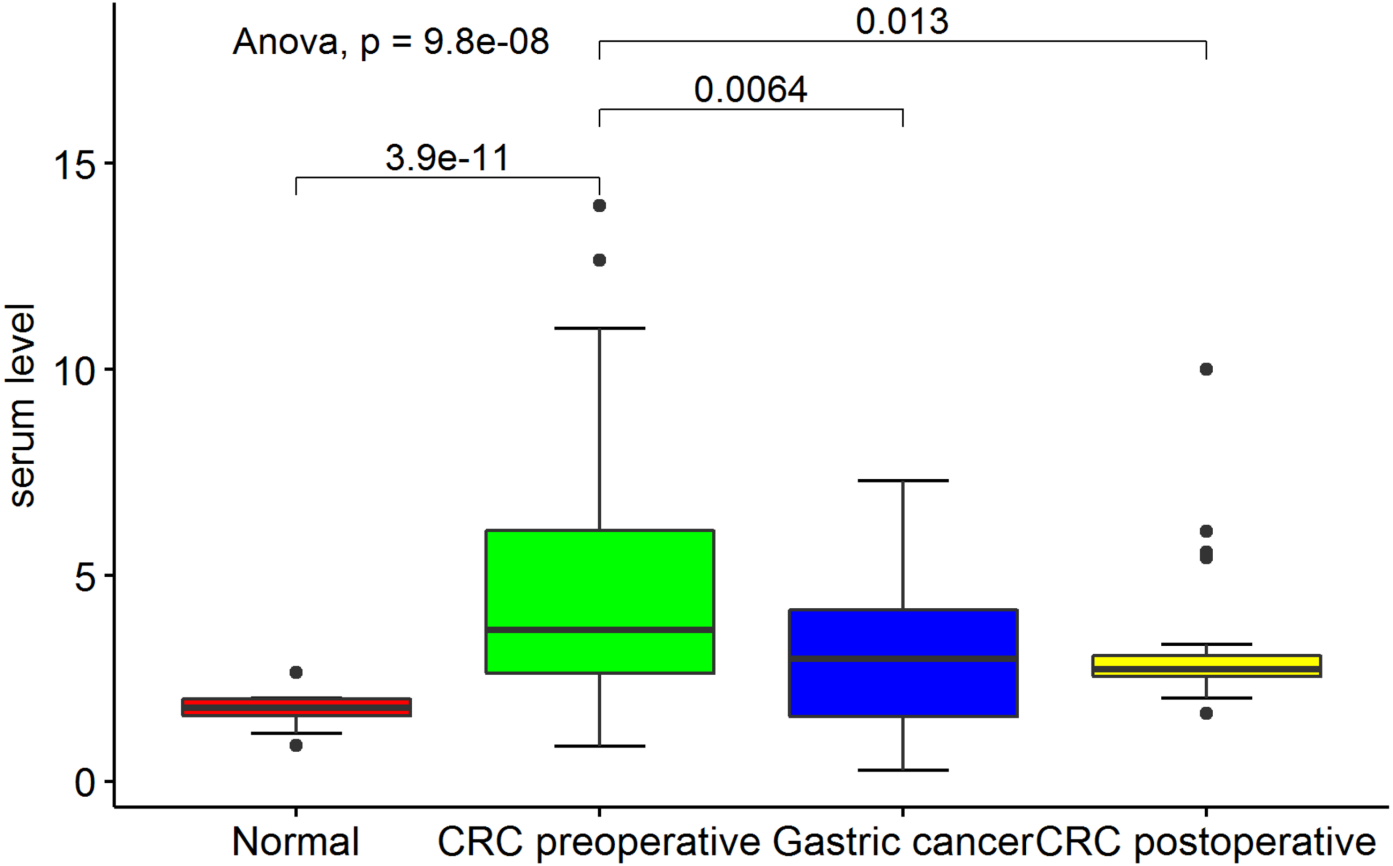
Comparison of serum SPINK4 level in preoperative- and postoperative- CRC, gastric cancer and healthy controls.

Serum was finally collected from 70 preoperative CRC patients, 30 postoperative CRC patients, 30 gastric cancer patients, and 30 healthy controls. The demographics of the participants are listed in Table 1. The serum SPINK4 level was highest in preoperative CRC patients compared with postoperative CRC patients, gastric cancer patients, and healthy controls (*p* < 0.05). There were no significant differences in the serum SPINK4 level among postoperative CRC patients, gastric cancer patients, and healthy controls (*p* > 0.05) (Fig. 1).

**Table 1:** Demographics of included subjects.

	CRC preoperative	CRC postoperative	Gastric cancer	Healthy controls
*n*	70	30	30	30
Age	57.46 ± 12.54	58.57 ± 12.76	56.77 ± 13.28	54.37 ± 11.8
Gender				
Male	45	20	18	20
Female	25	10	12	10
T stage				–
T1+T2	3	0	13	
T3+T4	67	30	17	
N stage				–
N0	3	0	10	
N1+N2	67	30	20	
M stage				–
M0	24	8	15	
M1	46	22	15	
				–
Grade				
Well differentiated	15	8	12	
Moderately differentiated	2	4	10	
Poorly differentiated	53	8	8	
Location			–	–
Colon	52	22		
Rectal	18	8		
CEA	14.14(8.74–180.64)	6.67(2.22–66.22)	2.64(1.76–3.54)	15.05(12.1–22.2)
CA125	27.13(19.25–35.43)	12.1(7.8–17.62)	12.88(8.52–20.78)	16.57(12.1–22.2)
CA153	23.04(19.22–27.75)	10.55(9.23–17.7)	10.16(8.28–13.5)	11.40(7.58–14.2)
CA199	26.61(19.04–119.73)	14.4(4.73–58.98)	8.18(5.86–14.77)	8.70(6.33–12.90)
SPINK4	3.72(2.09–4.67)	2.29(1.55–3.56)	2.68(2.45–3.39)	2.76 (2.60–2.88)

**Notes.**

Comparison of continuous data using variance or nonparametric methods; comparison of discrete distributions using Chi-square test.

### Association of preoperative serum SPINK4 level with clinical features in CRC

**Figure 2:**
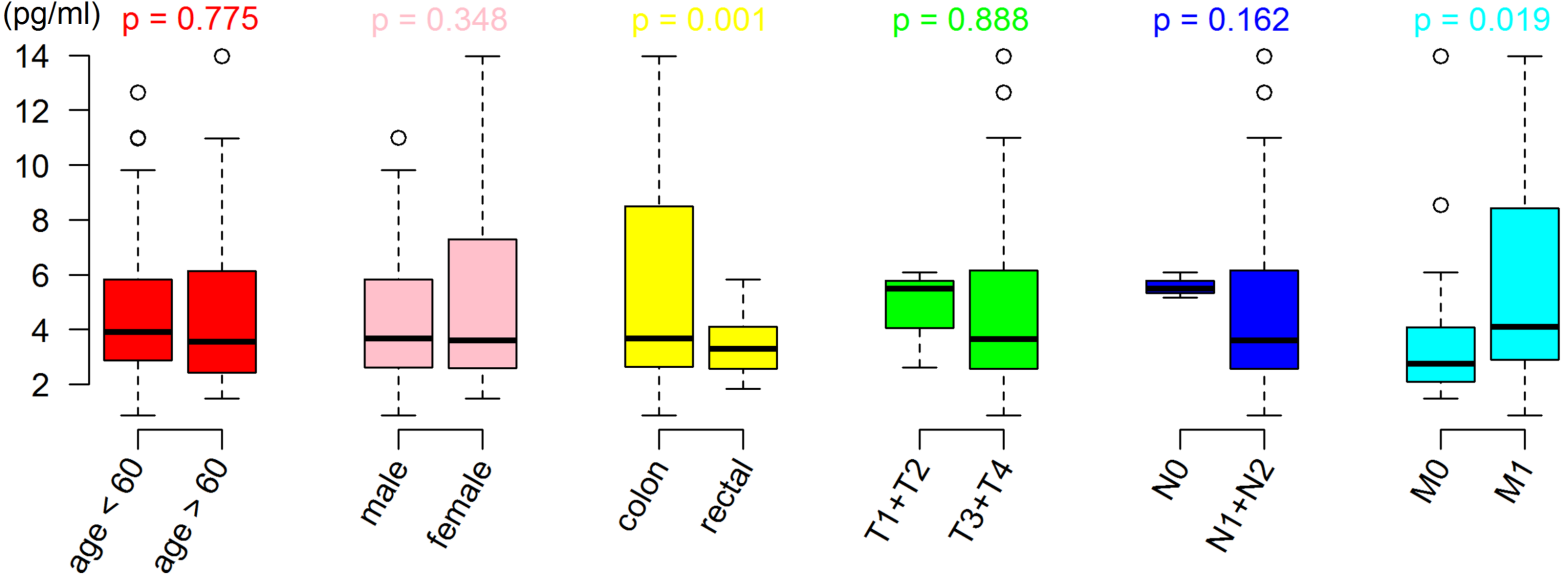
Difference of serum SPINK4 level in CRC with different clinical features. The differences among groups were compared using Student’s *t*-test.

The results showed that CRC patients with distant metastasis (M1 stage) had a higher serum SPINK4 level than those without distant metastasis (M0 stage). In addition, the serum SPINK4 level in patients with colon cancer was significantly elevated compared with patients with rectal cancer, indicating that metastatic status and location of tumor affect the serum SPINK4 level. However, there were no significant differences according to sex, T stage, N stage, and histologic grade (see Table 2 and Fig. 2). Furthermore, the correlation analysis failed to show the correlation of SPINK4 with CEA, CA125, CA153, and CA199 in CRC patients (Spearman’s rank correlation test, all *p* > 0.05) (Fig. S1)

**Table 2:** Association of preoperative serum SPINK4 level with clinical features in CRC.

	Expression value	*p*-value
Age (years)		0.759
>60 years	5.03 ± 3.06	
≤ 60 years	4.79 ± 3.41	
Gender		0.366
Male	5.16 ± 3.50	
Female	4.48 ± 2.68	
T stage		0.882
T1+T2	4.73 ± 1.85	
T3+T4	4.92 ± 3.28	
N stage		0.163
N0	5.58 ± 0.47	
N1+N2	4.88 ± 3.29	
M stage		0.021
M0	3.79 ± 2.72	
M1	5.53 ± 3.33	
Grade		0.589
Low	5.40 ± 4.09	
High+ middle	4.78 ± 2.97	
Location		0.002
Colon	5.42 ± 3.58	
Rectal	3.54 ± 1.16	

**Notes.**

The differences among groups were compared using Student’s *t*-test.

### Diagnostic value of serum SPINK4 level in CRC patients

**Figure 3:**
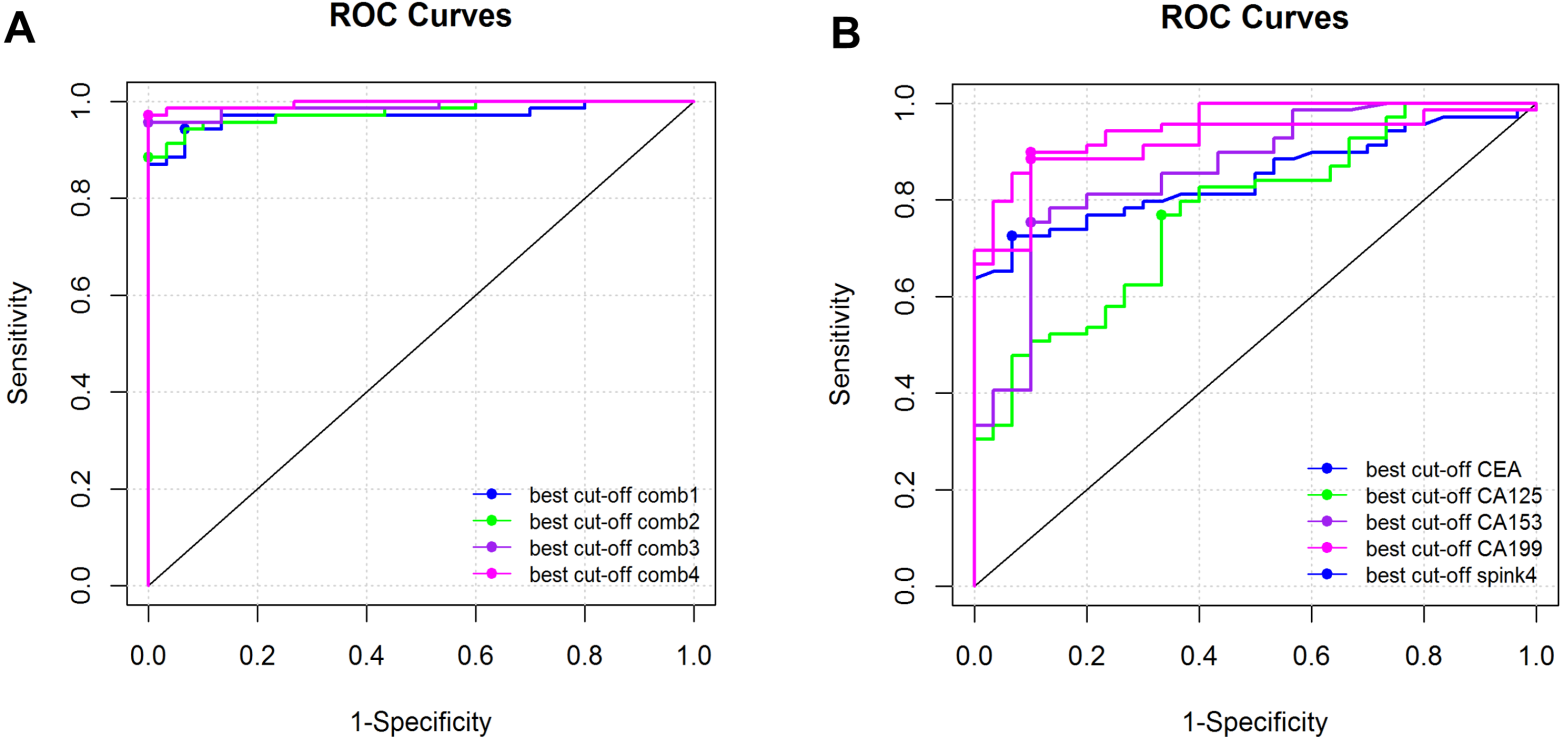
Diagnostic value of serum biomarkers in CRC. (A) Diagnostic value of serum SPINK4, CEA, CA125, CA153, CA199 in CRC. (B) Diagnostic value of serum SPINK4 combined with CEA, CA125, CA153 or CA199 in CRC. The data was analyzed using the receiver operating characteristic curve and areas under the curve with 95% confidence interval.

Using the sera of healthy people as controls, we analyzed the diagnostic value of serum SPINK4 in CRC. The results showed that serum SPINK4 had a higher diagnostic value in CRC, with an AUC of 0.919 (0.864–0.973), sensitivity and specificity of 0.886 and 0.900, respectively, and cut-off value of 2.065, which was better than the diagnostic value of serum CEA, CA125, CA153, and CA199. The combination of SPINK4 with CEA, CA125, CA153, and CA199 could increase the diagnostic value in CRC (see Table 3 and Fig. 3).

**Table 3:** Diagnostic value of SPINK4 and combination with other tumor biomarkers.

	Cut-off	Sensitivity	Specificity	AUC
SPINK4	2.065	0.886	0.900	0.919
CEA	3.260	0.667	0.724	0.845
SPINK4+CEA	–	0.967	0.800	0.970
SPINK4+CEA+ CA125	–	0.974	0.884	0.977
SPINK4+CEA+ CA125+ CA153	–	0.977	0.956	0.988
SPINK4+CEA+ CA125+ CA153+ CA199	–	0.979	0.971	0.996

### Prognostic value of serum SPINK4 in CRC patients

**Figure 4:**
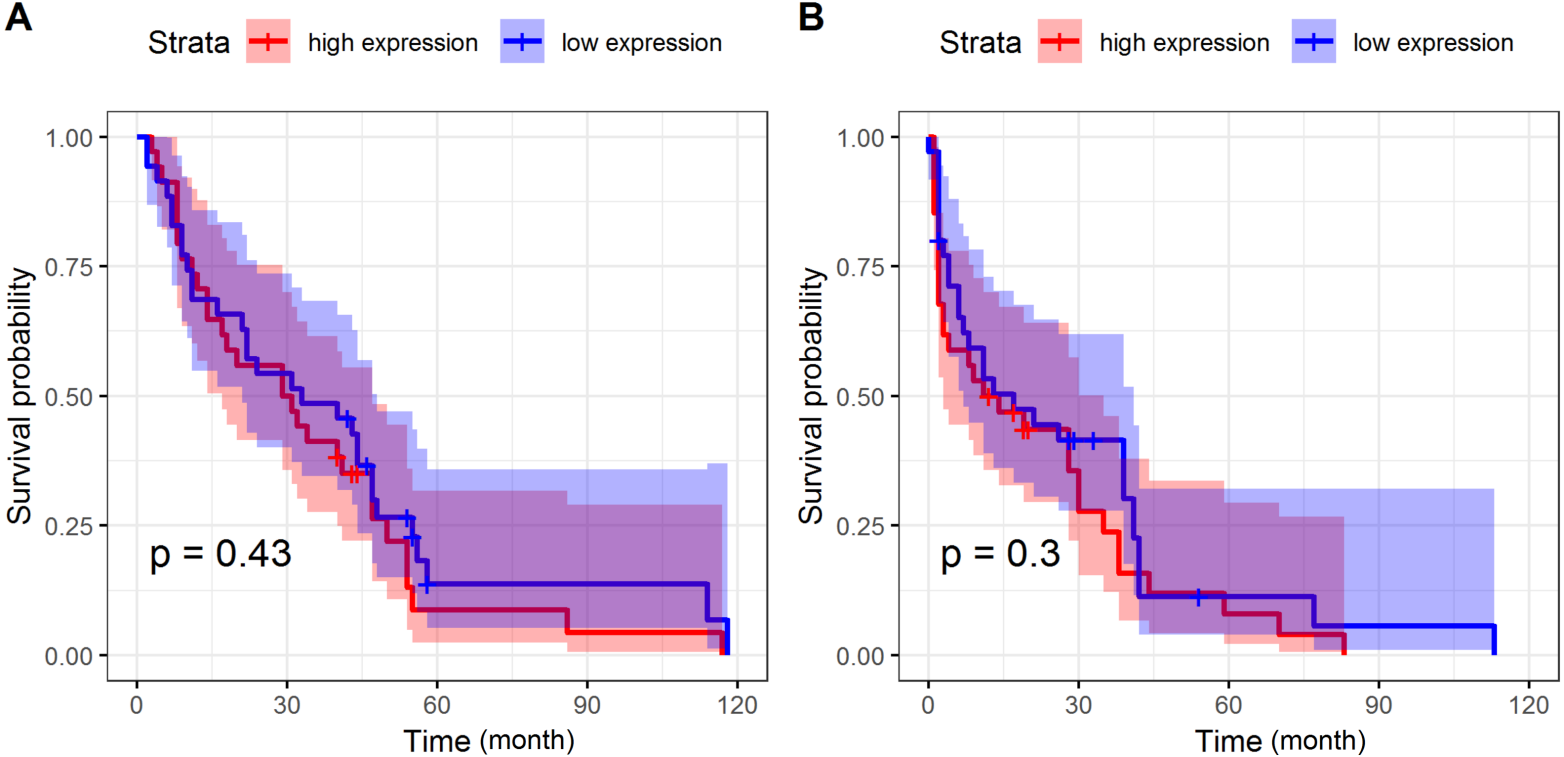
Prognostic value of serum SPINK4 in CRC patients. (A) Kaplan-Meier curve of OS of CRC patients with serum SPINK4 level. (B) Kaplan-Meier curve of DFS of CRC patients with serum SPINK4 level. Survival curves were drawn by the Kaplan-Meier method and compa

The follow-up of CRC patients in the present study lasted for 22 (0–133) months. Using the median value as cut-off, we divided the CRC patients into SPINK4 high- and low-expression groups. The survival analysis showed no significant difference between patients with high and low expressions of SPINK4 regarding OS and DFS, suggesting that serum SPINK4 might not act as a predictor among CRC patients (Fig. 4).

## Discussion

To date, the evidence supporting the usage of CEA, CA125, and CA199 in the diagnosis and prognosis of CRC has been insufficient. In the present study, the serum SPINK4 level was much higher in preoperative CRC patients than in postoperative CRC patients, gastric cancer patients, and healthy controls, suggesting that serum SPINK4 was specifically increased in CRC and decreased after resection of CRC. We also found that the serum SPINK4 level was associated with CRC location and metastasis. In addition, the serum SPINK4 level performed better in the diagnosis of CRC than conventional serum indicators. However, we failed to show the predictive value of serum SPINK4 on the OS and DFS of CRC patients, indicating that serum SPINK4 was not associated with the prognosis of CRC patients. Taken together, these results indicate that serum SPINK4 level could serve as an indicator in the diagnosis of CRC patients.

The *SPINK* gene is located on chromosome 9p13.3 and resides within a linkage region (9p21-13). To date, although the role of SPINK4 has been implied in some diseases, knowledge of SPINK4 in CRC remains limited. In previous reports, SPINK4 was found to be significantly upregulated in intestinal epithelial cells in active celiac disease (Pietz et al., 2017). In celiac disease, expression of SPINK4 was at its highest in untreated patients and dropped sharply upon commencement of a gluten-free diet; however, genetic association tests failed to show a difference between extended case/control cohorts regarding the expression of SPINK4 (Wapenaar et al., 2007). SPINK4 was also implicated in inflammatory bowel disease (IBD); Brenna et al. (2013) found that SPINK4 was remarkably enhanced in the colon mucosa of an IBD rat model compared with normal rats. Moreover, SPINK4 has also been identified as a risk locus for ulcerative colitis (Hasler et al., 2012) and Barrett’s esophagus (Owen et al., 2018). To date, only one study has explored the role of SPINK4 in colon cancer cells. Noah et al. (2010) showed that the goblet cell genes *AGR2, MUC2, and RETLNB*, and *SPINK4* were activated in LS174T colon cancer cells treated with a Notch/*γ*-secretase inhibitor, while inhibited SPDEF could repress the expression of these genes. These results suggested that SPINK4 might be involved in the mechanism by which SPDEF inhibits colon cancer cells proliferation. Collectively, recent studies demonstrated that SPINK4 was closely related to gastrointestinal diseases, but its role in CRC was largely unknown.

Currently, CEA, CA125, CA153, and CA199 are recommended as serum tumor biomarkers in cancers for tumor detection and monitoring response to therapy. Usually, CEA is used to diagnose CRC, and CA199 is used to diagnose pancreatic cancer (Hui, Rixv & Xiuying, 2015; Shi et al., 2010). However, a high serum level of CEA is not a specific indicator of CRC; other diseases, such as IBD and pancreatitis may also present with high serum levels of CEA. In this study, we found that the serum SPINK4 level was especially increased in preoperative CRC patients and reduced after tumor resection. It was also closely related to distant metastasis, suggesting that it could be used to monitor the response to therapy in CRC. The AUC value revealed that SPINK4 had a higher diagnostic value compared with CEA, CA125, CA153, or CA199, showing that the serum SPINK4 level could be used to aid the diagnosis of CRC when other serum biomarkers failed to diagnose CRC. This study also tested the diagnostic value of the combination of SPINK4 with other biomarkers, and the results showed that the combination of SPINK4 with 2 biomarkers could achieve high sensitivity and specificity (both over 90%). This result indicated that using a panel of SPINK4 with other biomarkers could achieve better diagnostic value for CRC diagnosis

With regards to the prognostic value of serum biomarkers in CRC, although some studies have reported that CEA could be used to predict the survival of CRC patients (Tsai et al., 2016), there were also inconsistent results (Song et al., 2018), which suggested that there was no definite evidence that CEA could act as a reliable predictor of the prognosis of CRC. Several serum biomarkers were also reported to have better prognostic value in CRC. Using a meta-analysis method, IL-6, platelet- lymphocyte ratio (PLR), and neutrophil-lymphocyte ratio (NLR) were able to predict the prognosis of CRC, with a hazard ratio (HR) of 1.76, 1.89, and 1.56 for OS, and 2.97, 1.49, and 1.92 for DFS, respectively (Xu et al., 2016; Zhang et al., 2017). TNM stage is the most common system used to assist in treatment decisions, and to predict the prognosis of cancer patients. However, some studies have pointed out that TNM staging needs to be modified in order to improve the predictive value in CRC (Li et al., 2014; Li et al., 2016). In the present study, we did not find that serum SPINK4 was associated with the OS or DFS of CRC patients. There are at least two reasons that may explain the result. First, the follow-up time was shorter in the present study; almost half of the patients were followed up for less than 10 months. Second, the sample size was small, which would undermine the statistical power to detect significant differences. Therefore, further studies with larger cohorts and a longer follow-up time are necessary to examine the prognostic value of serum SPINK4.

Although this study showed that the serum SPINK4 level was closely related to CRC and had a higher diagnostic value in CRC, some limitations should be noted. First, the sample size of the study was relatively small, and we only included CRC and gastric cancer patients; other gastrointestinal cancers should be included. Second, some risk factors for CRC, including smoking, heavy alcohol use, and lifestyle were not analyzed in this study, which might affect the reliability of the results. Third, most of the CRC patients included this study were at an advanced disease stage, and this study was retrospective, which may lead to selection bias. Fourth, the follow-up time of the patients was shorter, which might affect the predictive value. Fifth, the present study focused on the value of the serum level of SPINK4 in CRC; however, due to a lack of tissue samples, we could not test its expression in tissue, and therefore could not further verify the value of SPINK4 in CRC. Therefore, future studies that address the aforementioned limitations of our study are needed in order to verify the diagnostic and prognostic value of serum SPINK4 in CRC.

## Conclusion

The present study demonstrated that the serum SPINK4 level was elevated in preoperative CRC, was decreased after resection of CRC, and was associated with the location and distant metastasis of CRC. Serum SPINK4 level has a high diagnostic value in CRC but may not be a prognostic indicator for CRC patients.

##  Supplemental Information

10.7717/peerj.6679/supp-1 Data S1 Raw materials for this study The serum level of SPINK4 in 70 preoperative CRC patients, 30 postoperative CRC patients, 30 gastric cancer patients, and 30 healthy controls

10.7717/peerj.6679/supp-2 Figure S1 The correlation between SPINK4 and other serum biomarkers in CRC

## Abbreviation

CRC: colorectal cancer
SPINK4: serine peptidase inhibitor, Kazal type 4
OR: odds ratio
HR: hazard ratio
ROC: Receiver operating characteristic curve
AUC: areas under the curve
CEA: carcinoembryonic antigen
CA199: carbohydrate antigen 19-9
OS: overall survival
DFS: disease-free survival

## Additional Information and Declarations

### Data Availability

The following information was supplied regarding data availability: The raw data is available as File S1.
