# Supplementary figures and images for: Association and diagnostic value of serum SPINK4 in colorectal cancer
Source: PeerJ. 2019 Apr 4;7:e6679. doi: 10.7717/peerj.6679 (PMC6451835; doi:10.7717/peerj.6679)

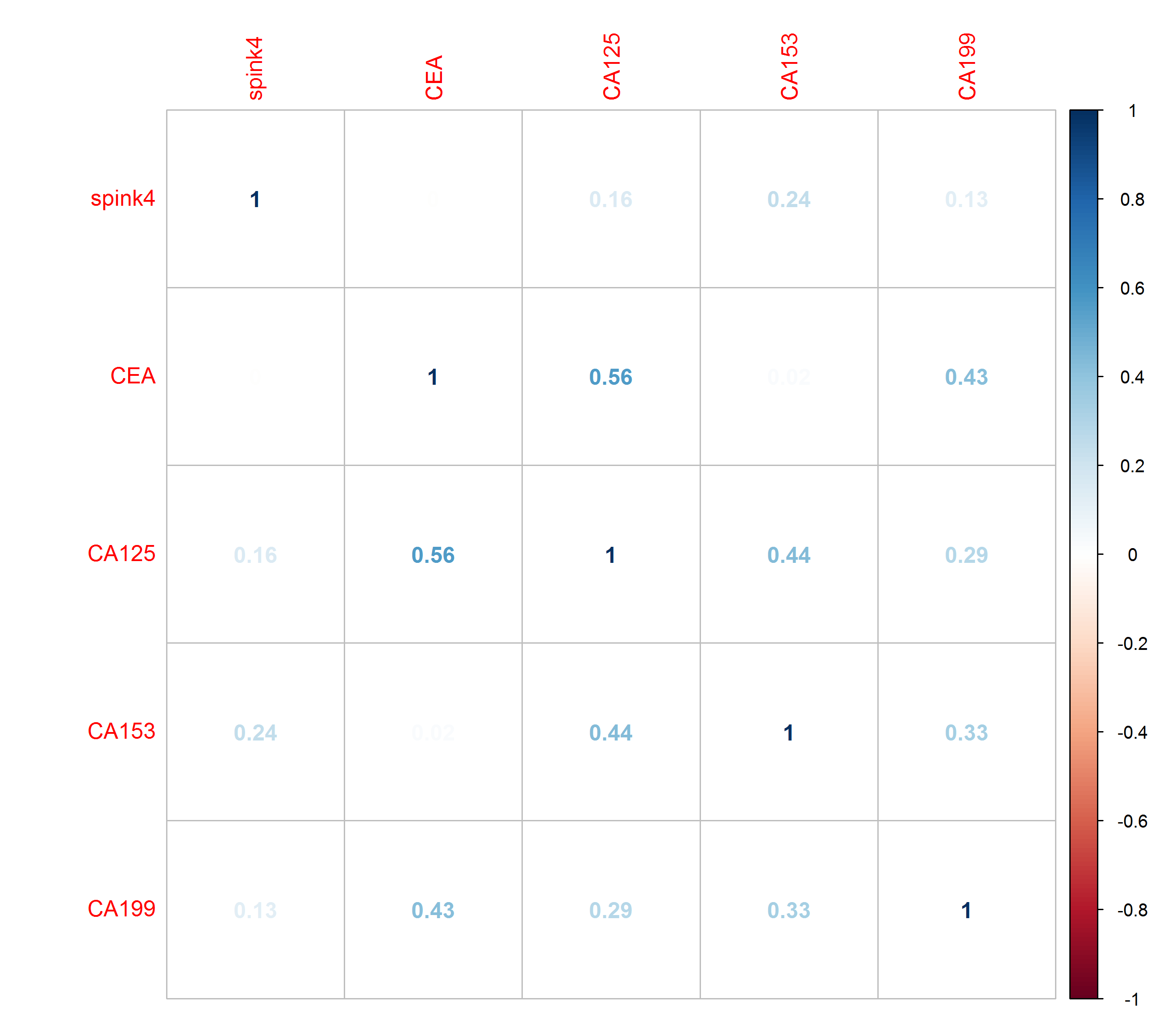

Supplement: Figure S1 [file peerj-07-6679-s002.png]
